# Non-invasive stroke volume measurement and passive leg raising predict volume responsiveness in medical ICU patients: an observational cohort study

**DOI:** 10.1186/cc7955

**Published:** 2009-07-08

**Authors:** Steven W Thiel, Marin H Kollef, Warren Isakow

**Affiliations:** 1 Pulmonary and Critical Care Division, Washington University School of Medicine, Campus Box 8052, 660 South Euclid Avenue, St. Louis, MO 63110, USA

## Abstract

**Introduction:**

The assessment of volume responsiveness and the decision to administer a fluid bolus is a common dilemma facing physicians caring for critically ill patients. Static markers of cardiac preload are poor predictors of volume responsiveness, and dynamic markers are often limited by the presence of spontaneous respirations or cardiac arrhythmias. Passive leg raising (PLR) represents an endogenous volume challenge that can be used to predict fluid responsiveness.

**Methods:**

Medical intensive care unit (ICU) patients requiring volume expansion were eligible for enrollment. Non-invasive measurements of stroke volume (SV) were obtained before and during PLR using a transthoracic Doppler ultrasound device prior to volume expansion. Measurements were then repeated following volume challenge to classify patients as either volume responders or non-responders based on their hemodynamic response to volume expansion. The change in SV from baseline during PLR was then compared with the change in SV with volume expansion to determine the ability of PLR in conjunction with SV measurement to predict volume responsiveness.

**Results:**

A total of 102 fluid challenges in 89 patients were evaluated. In 47 of the 102 fluid challenges (46.1%), SV increased by ≥15% after volume infusion (responders). A SV increase induced by PLR of ≥15% predicted volume responsiveness with a sensitivity of 81%, specificity of 93%, positive predictive value of 91% and negative predictive value of 85%.

**Conclusions:**

Non-invasive SV measurement and PLR can predict fluid responsiveness in a broad population of medical ICU patients. Less than 50% of ICU patients given fluid boluses were volume responsive.

## Introduction

Circulatory insufficiency is a common clinical problem faced by physicians caring for critically ill patients. The decision to employ volume expansion (VE) in these patients is complicated [1]. If a patient is preload responsive, then VE improves cardiac output (CO). Early resuscitation protocols that include fluid therapy can be life saving early in the course of sepsis [2,3]. However, in a preload unresponsive patient, volume administration has no hemodynamic benefit. Liberal volume resuscitation can exacerbate pulmonary edema, precipitate respiratory failure, prolong mechanical ventilation times, and contribute to the development of intra-abdominal hypertension [4-6]. Prior studies have shown positive fluid balance to correlate with reduced survival [7-9]. In addition, prospective studies have shown that less than 50% of critically ill patients respond to the fluid boluses that are deemed necessary by treating clinicians [10-14]. A simple, non-invasive bedside test to determine volume responsiveness that would assist clinicians in facing this daily dilemma would have significant utility.

Passive leg raising (PLR) is a simple maneuver used for generations as an initial intervention for patients in shock. This procedure rapidly returns 150 to 200 ml of blood from the veins of the lower extremities to the central circulation [15]. As a result of increased ventricular preload, the CO is augmented according to the degree of preload reserve, and rapidly reversed when the legs are returned to a horizontal position. PLR therefore constitutes a reversible volume challenge during which hemodynamic changes can be measured [16].

The aim of our study was to determine if noninvasive stroke volume (SV) measurement could be used in conjunction with PLR to predict the hemodynamic response to VE.

## Materials and methods

### Patients

This study was conducted at Barnes-Jewish Hospital, a university-affiliated, urban teaching hospital. The study was approved by the Washington University School of Medicine Human Studies Committee. As the protocol was considered part of routine practice, informed consent was waived. Patients were informed that they participated in this study. Patients were enrolled from the medical intensive care unit (ICU), and any patient requiring VE as determined by the ICU attending physician was eligible for enrollment. No specific criteria for circulatory insufficiency were required for study entry. However, the decision of the ICU attending to administer fluid was based on clinical signs of inadequate tissue perfusion (e.g. escalating vasopressor requirement, decreasing urine output, etc.) and his/her clinical impression that the patient should be given a trial of volume expansion. Exclusion criteria included known aortic or pulmonary valve disease, known ascending aortic aneurysm, or contraindication to PLR for any reason.

### Data collection

Stroke volume measurements were taken using a non-invasive, transthoracic Doppler ultrasound device (USCOM^®^; Uscom Ltd., Sydney, Australia). All measurements were performed by a single investigator (ST) following training on the device. Each study measurement was taken in accordance with a previously described protocol designed to optimize accuracy and reliability [17]. The device used directly measures the blood flow through either the aortic or pulmonary valves. For each patient studied, both positions were attempted and the location that resulted in the best signal was used.

**Figure 1:**
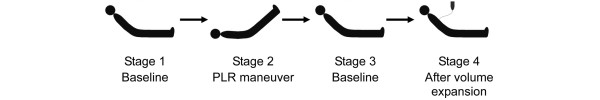
Patient positioning during the four stages of measurement. After each change in position, two minutes elapsed before readings were recorded. The angle of elevation of the head or legs was 45 degrees. The patient's position was not changed between stages three and four.

Study measurements were taken in four stages (Figure 1). In stage one the patient was placed in a semi-recumbent position with the head elevated at 45 degrees. In stage two, the patient was positioned supine with the legs straight and elevated at 45 degrees for two minutes. Stage three readings were taken two minutes after the patient was returned to the baseline position, and stage four immediately following VE. Calibrated automatic bed elevation (using standard ICU beds) was used to move the patient between stages.

Products for VE varied according to the order of the attending physician and included normal saline, Ringer's lactate and hetastarch. The volume administered in each case was at least 500 ml, and was given as a pressurized rapid infusion.

Vasopressor doses and ventilator settings were not changed at any time while a patient was being studied. Lower extremity compression devices were removed prior to the initial readings. Study measurements were recorded before, during, and after PLR and after VE throughout the stages described above.

### Definition of volume responsiveness

Patients were classified according to their hemodynamic response to VE. Responders had a SV increment of at least 15% in response to VE (an increase in SV from stage one to stage four), while non-responders had a SV increase of less than 15%. Cutoff values of 10% to 15% have been previously used as representing a significant change in SV and cardiac index in similar studies [1,16,18-20], and a 15% change was reported as a significant difference between two measures of CO by thermodilution [21].

### Statistical analysis

Continuous data are expressed as mean ± standard deviation. The Student's t-test was used for comparisons made between parametric data, and nonparametric data were analyzed with the Mann-Whitney U test. For categorical variables, chi-squared or Fisher's exact tests were used to test for differences between groups. The areas under receiver operating characteristic (ROC) curves are expressed as the area ± standard error, and were compared using the Hanley-McNeil method [22]. All tests were two-tailed, and a *P *value of less than 0.05 was pre-determined to be statistically significant. Where applicable, the Bonferroni multiplicity adjustment to the *P *value considered statistically significant is given [23,24]. Analyses were performed using the SPSS^© ^version 11.0.1 software package (SPSS Inc., Chicago, IL, USA).

## Results

### Patient characteristics

A total of 102 volume challenges in 89 consecutive patients were evaluated. One patient had three studies performed, while the remaining patients with more than one study had two studies each. Repeat studies performed on the same patient were separated in time by at least 24 hours. Thirteen additional patients were examined, although either they were unable to tolerate the procedure (three patients), unable to cooperate due to confusion or delirium (six patients), or satisfactory Doppler signals could not be obtained (four patients).

Stroke volume increased by 15% or more in 47 (46.1%) instances (responders), and by less than 15% in 55 (53.9%) instances (non-responders). For the purposes of data analysis, each volume challenge was considered an independent observation regardless of whether it was part of multiple studies performed on the same patient.

The resulting pool of volume challenges were performed on patients who were aged 59.4 ± 15.1 years, with 58 (56.9%) men and 44 (43.1%) women. Fifty-nine (57.8%) patients were receiving vasopressor support, 67 (65.7%) were mechanically ventilated, with 14 (20.9%) of those fully accommodated to the ventilator, and their Acute Physiology and Chronic Health Evaluation II score was 18.5 ± 6.1. The time elapsed between ICU admission and study entry was 61.7 ± 106.2 hours. Cardiac arrhythmias were present in 18 (17.5%) patients (atrial fibrillation in eight, premature ventricular beats in six, and premature atrial beats in four). The patient characteristics are summarized in Table 1.

**Table 1:** Patient characteristics and etiology of circulatory insufficiency

	All	Responders	Non-responders	*P*
Age (years)	59.4 ± 15.1	56.1 ± 13.5	62.2 ± 15.9	0.04
Sex, n (%)				
Male	58 (56.9%)	30 (63.8%)	28 (50.9%)	0.19
Female	44 (43.1%)	17 (36.2%)	27 (49.1%)	
BMI (kg/m^2^)	31.0 ± 11.5	31.6 ± 11.7	30.5 ± 11.5	0.66
Admitted from, n (%)				
ED	49 (48.0%)	23 (48.9%)	26 (47.3%)	0.87
Other hospital	17 (16.7%)	7 (14.9%)	10 (18.2%)	0.79
Ward	36 (35.3%)	17 (36.2%)	19 (34.5%)	0.86
Time since ICU admission (hours)	61.7 ± 106.2	52.2 ± 95.9	69.9 ± 114.6	0.40
APACHE II score	18.5 ± 6.1	17.8 ± 5.9	19.2 ± 6.2	0.29
Mechanical ventilator	67 (65.7%)	34 (72.3%)	33 (60.0%)	0.19
Vasopressor support	59 (57.8%)	27 (57.4%)	32 (58.2%)	0.94
Norepinephrine dose (mcg/kg/min) *	0.17 ± 0.15	0.16 ± 0.17	0.17 ± 0.14	0.88
Fluid administered since onset of circulatory	6277 ± 7180	5775 ± 5829	6713 ± 8208	0.52
Insufficiency (ml)				
Arrhythmia present	18 (17.6%)	3 (6.4%)	15 (27.3%)	0.008
Clinical diagnosis **				
Sepsis	62 (60.8%)	27 (57.4%)	35 (63.6%)	0.52
Cardiogenic shock	4 (3.9%)	1 (2.1%)	3 (5.5%)	0.62
Hypovolemia	20 (19.6%)	10 (21.3%)	10 (18.2%)	0.69
Brain injury	1 (1.0%)	0 (0%)	1 (1.0%)	
Toxic ingestion	1 (1.0%)	0 (0%)	1 (1.0%)	
Other	2 (2.0%)	1 (1.0%)	1 (1.0%)	
Unknown	12 (11.8%)	8 (17.0%)	4 (7.3%)	0.22

The *P *values given are for comparisons between the responders and non-responders.

* All but two patients who required vasopressor support were on norepinephrine alone. Those patients (both non-responders) are not included in this calculation.

** Diagnostic impression of the attending physician.

APACHE = acute physiology and chronic health evaluation; BMI = body mass index; ED = emergency department; ICU = intensive care unit.

### Effects of PLR and volume expansion

The initial hemodynamic measurements are summarized in Table 2. The responders had a significantly lower initial SV (68 ± 25 ml vs. 87 ± 30 ml, *P*<0.001 compared with the non-responders, although the CO (6.8 ± 2.5 L/min vs. 8.0 ± 2.9 L/min, *P *= 0.03), corrected flow time (FTc; 363 ± 70 ms vs. 398 ± 66 ms, *P *= 0.01), mean arterial pressure (MAP; 68 ± 13 mmHg vs. 74 ± 14 mmHg, *P *= 0.03), and heart rate (101 ± 20 beats/min vs. 93 ± 20 beats/min, *P *= 0.06) were not different between the groups (Bonferroni adjusted level of significance for all comparisons 0.01).

**Table 2:** Initial hemodynamic readings taken in stage one

	All	Responders	Non-responders	*P*
Stroke volume (ml)	79 ± 29	68 ± 25	87 ± 30	< 0.001
Cardiac output (L/min)	7.4 ± 2.8	6.8 ± 2.5	8.0 ± 2.9	0.03
Corrected flow time (ms)	382 ± 70	363 ± 70	398 ± 66	0.01
Mean arterial pressure (mmHg)	71 ± 13	68 ± 13	74 ± 14	0.03
Pulse pressure (mmHg)	45 ± 15	42 ± 14	48 ± 15	0.02
Heart rate (beats/min)	96 ± 20	101 ± 20	93 ± 20	0.06
Central venous pressure				
Number of observations	59 (57.8%)	25 (53.2%)	34 (61.8%)	0.38
Value (mmHg)	7.9 ± 4.8	7.8 ± 4.9	8.1 ± 4.8	0.80

The *P *values given are for comparisons between the responders and non-responders. Except for the comparison of the central venous pressure, the Bonferroni adjusted level of significance for all *P *values shown is 0.01.

The hemodynamic readings taken throughout the four stages of measurements are summarized in Table 3. For the responders, PLR induced a significant increase in SV (68 ± 25 ml vs. 82 ± 30 ml, *P *= 0.001), but the CO (6.8 ± 2.5 L/min vs. 8.0 ± 2.8 L/min, *P *= 0.03), FTc (363 ± 70 ms vs. 380 ± 68 ms, *P *= 0.22), MAP (68 ± 13 mmHg vs. 72 ± 11 mmHg, *P *= 0.11), heart rate (101 ± 20 beats/min vs. 99 ± 21 beats/min, *P *= 0.64), and pulse pressure (42 ± 14 mmHg vs. 45 ± 14 mmHg, *P *= 0.23) were unchanged (Bonferroni adjusted level of significance for all comparisons 0.01). The increase in SV was completely reversed when the patient was returned to the semi-recumbent position.

**Table 3:** Hemodynamic readings taken throughout the four stages of measurement

	Stage 1	Stage 2	*P*_2,1_	Stage 3	*P*_3,1_	Stage 4	*P*_4,1_
**Responders**							
SV (ml)	68 ± 25	82 ± 30	0.001	70 ± 26	0.76	86 ± 31	0.004
SV % change from stage 1		21.0 ± 12.5		2.4 ± 7.8		26.3 ± 14.2	
CO (L/min)	6.8 ± 2.5	8.0 ± 2.8	0.03	6.9 ± 2.6	0.89	8.3 ± 3.1	0.009
FTc (ms)	363 ± 70	380 ± 68	0.22	356 ± 59	0.62	393 ± 66	0.03
MAP (mmHg)	68 ± 13	72 ± 11	0.11	70 ± 11	0.41	71 ± 16	0.38
Heart rate (beats/min)	101 ± 20	99 ± 21	0.64	100 ± 21	0.81	99 ± 20	0.61
Pulse pressure (mmHg)	42 ± 14	45 ± 14	0.23	45 ± 13	0.30	49 ± 16	0.02
CVP (mmHg)	7.8 ± 4.9					9.9 ± 3.9	0.10
**Non-responders**							
SV (ml)	87 ± 30	91 ± 33	0.58	88 ± 30	0.99	90 ± 31	0.62
SV % change from stage 1		3.2 ± 10.4		0.3 ± 5.9		3.5 ± 8.6	
CO (L/min)	8.0 ± 2.9	8.4 ± 3.5	0.46	7.9 ± 2.9	0.97	8.2 ± 3.1	0.71
FTc (ms)	398 ± 66	404 ± 78	0.66	399 ± 68	0.89	405 ± 68	0.58
MAP (mmHg)	74 ± 14	74 ± 16	0.95	73 ± 14	0.72	74 ± 16	0.97
Heart rate (beats/min)	93 ± 20	94 ± 21	0.84	93 ± 20	0.91	92 ± 20	0.75
Pulse pressure (mmHg)	48 ± 15	49 ± 17	0.97	49 ± 18	0.89	49 ± 19	0.83
CVP (mmHg)	8.1 ± 4.8					11.3 ± 5.5	0.01

Except for the comparison of the stage 1 and 4 CVP, the Bonferroni adjusted level of significance for all P values shown is 0.01.

CO = cardiac output; CVP = central venous pressure; FTc = corrected flow time; MAP = mean arterial pressure; SV = stroke volume.

In the non-responders, PLR did not induce a significant change in any of the hemodynamic values measured. The SV (87 ± 30 ml vs. 91 ± 33 ml, *P *= 0.58), CO (8.0 ± 2.9 L/min vs. 8.4 ± 3.5 L/min, *P *= 0.46), FTc (398 ± 66 ms vs. 404 ± 78 ms, *P *= 0.66), MAP (74 ± 14 mmHg vs. 74 ± 16 mmHg, *P *= 0.95), heart rate (93 ± 20 beats/min vs. 94 ± 21 beats/min, *P *= 0.84), and pulse pressure (48 ± 15 mmHg vs. 49 ± 17 mmHg, *P *= 0.97) remained unchanged during PLR.

**Figure 2:**
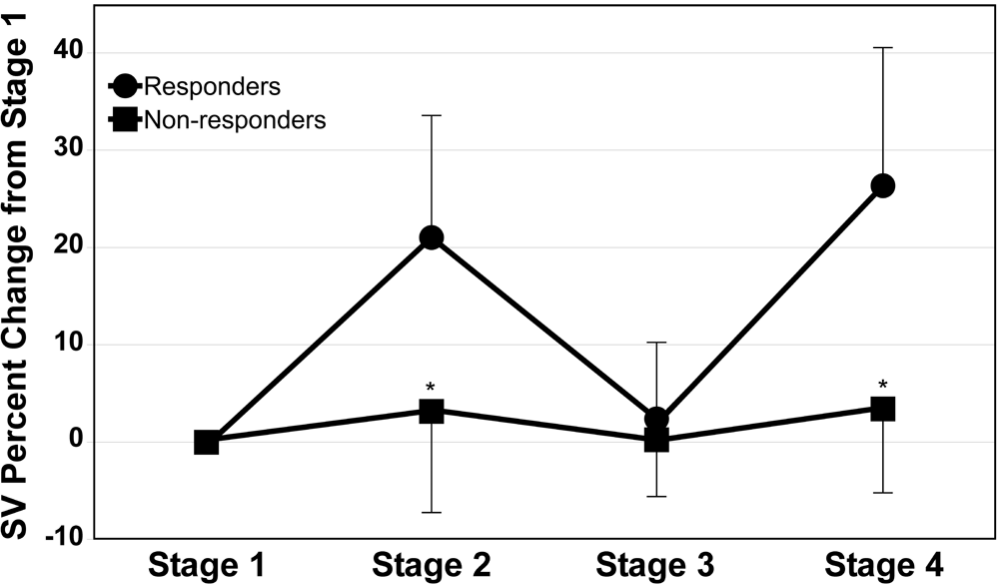
Stroke volume change by stage for responders and non-responders. Each measurement is represented as a percent change from the measurement taken during stage one (* *P *< 0.001, Bonferonni adjusted level of significance 0.01). SV = stroke volume.

The changes in SV compared with stage one induced by both PLR and VE were significantly higher in the responders compared with the non-responders. The SV increased in response to PLR in the responders and non-responders by 21.0% ± 12.5% and 3.2% ± 10.4%, respectively (*P*<0.001, Bonferroni adjusted level of significance 0.01; Figure 2). The SV increased in response to VE in the responders and non-responders by 26.3% ± 14.2% and 3.5% ± 8.6%, respectively (*P *< 0.001, Bonferroni adjusted level of significance 0.01). The PLR-induced increase in SV was reversed once the patient was taken out of the PLR position (Table 3).

### Central venous pressure

The initial central venous pressure (CVP) was not different between the groups of responders and non-responders (7.8 ± 4.9 mmHg vs. 8.1 ± 4.8 mmHg, *P *= 0.80; Table 2). Additionally, the change in CVP between stages one and four was not different between the responders and non-responders (2.1 ± 3.0 mmHg vs. 3.2 ± 2.3 mmHg, *P *= 0.13).

### Prediction of volume response

**Figure 3:**
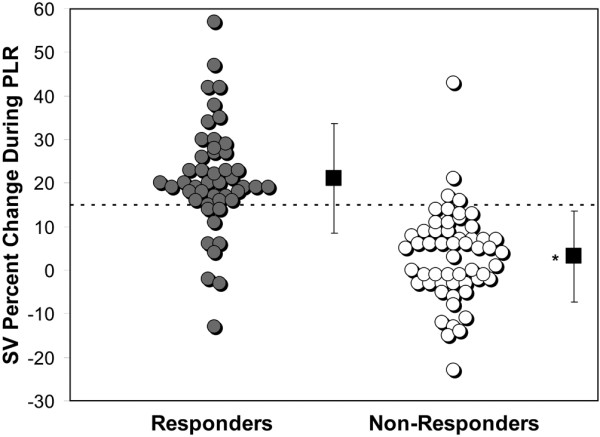
Individual percent change in stroke volume during passive leg raise for responders and non-responders. The dashed line represents the cutoff value of 15%. The squares represent the means with SD of the two groups (* *P *< 0.001, Bonferonni adjusted level of significance 0.01). PLR = passive leg raise; SV = stroke volume.

A SV increase induced by PLR of 15% or more predicted volume response with a sensitivity of 81%, specificity of 93%, positive predictive value of 91%, and a negative predictive value of 85% (Figure 3).

**Figure 4:**
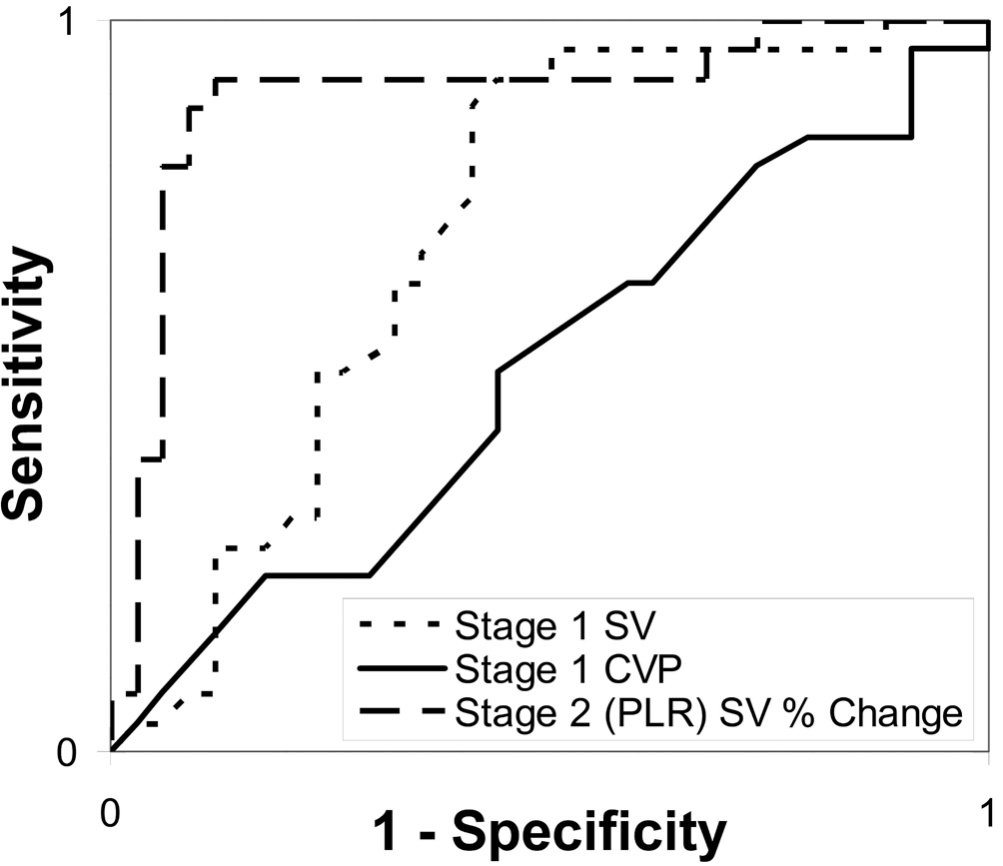
Receiver operating characteristic curves for predicting response to volume expansion. The dashed line represents the percent change in stroke volume (SV) during passive leg raise (PLR), the dotted line the stage one SV, and the solid line the stage one central venous pressure (CVP).

The area under the ROC curve for the percent change in SV during PLR predicting a response to VE was 0.89 ± 0.04. Other than the SV, no hemodynamic index significantly changed during PLR. However, several other indices were different, although not statistically significant, at baseline between the responders and non-responders. ROC curves for these initial measures predicting volume response were also constructed. Compared with the SV change during PLR these indices were inferior at differentiating the responders from the non-responders, and included the stage one SV (ROC curve area 0.70 ± 0.05, *P *= 0.001), CO (0.62 ± 0.06, *P *< 0.001), CVP (0.52 ± 0.08, *P *< 0.001), MAP (0.63 ± 0.06, *P *< 0.001), and FTc (0.65 ± 0.06, *P *< 0.001). The ROC curves for SV change with PLR and initial CVP and SV are shown in Figure 4.

### Repeatability of measurements

**Figure 5:**
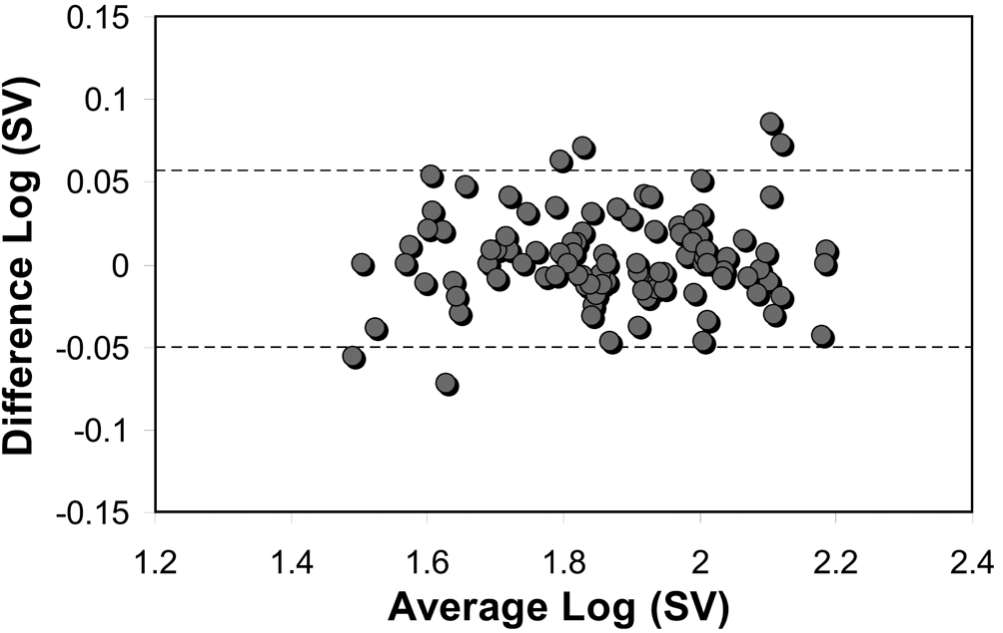
Bland-Altman plot of log-transformed difference against mean for paired stroke volume measurements from stages one and three. The dashed lines represent the log-transformed upper and lower limits of agreement (95% confidence interval for repeated measurements). SV = stroke volume.

A repeatability analysis was performed using the paired readings for stages one and three from each patient. The hemodynamic effects of PLR are transient and reversible, and vasoactive agents were not changed between these measurements. Therefore, it is expected that the readings from these stages would not be different and can be used to validate the use of a 15% change in SV as being significant. Using the method described by Bland and Altman [25] the upper and lower limits of agreement between stages one and three were 13.9% (95% confidence interval (CI) = 13.2% to 14.6%) and -10.9% (95% CI = -11.6% to -10.2%), respectively. The corresponding plot of the log-transformed SV difference against mean is shown in Figure 5.

## Discussion

Our study demonstrates that a completely non-invasive SV measurement in conjunction with PLR can predict the hemodynamic response to VE. In our relatively unselected population of medical ICU patients, the change in SV with PLR was the only hemodynamic index with significant predictive ability. The initial CVP was not different between the groups of responders and non-responders, and the change in CVP did not correlate with the change in SV following VE. A repeatability analysis revealed that a cutoff of 15% representing a significant change in SV is reasonable.

The ultrasound device used in this study has been previously evaluated for accuracy and reliability. Knobloch and colleagues studied 36 patients undergoing coronary revascularization with 180 paired CO and SV measurements using the USCOM^® ^and a pulmonary artery catheter (PAC) [26]. Good correlation was found for both CO and SV (correlation index 0.79, *P *< 0.01 and 0.95, *P *< 0.01, respectively), and a Bland-Altman analysis demonstrated a bias of 0.23 ± 1.01 L/min for the CO measurements. Chand and colleagues studied 50 patients following coronary artery bypass surgery and compared SV measurements obtained with the USCOM^® ^and the PAC [27]. The SV measurements demonstrated a bias of 1.0 ml (limits of agreement -1.5 ml to 3.5 ml) for aortic measurements and 1.6 ml (limits of agreement -0.21 ml to 3.4 ml) for pulmonary readings. Tan and colleagues examined 24 mechanically ventilated patients following cardiac surgery and compared 40 paired CO readings obtained by the USCOM ^® ^and the PAC [28]. The resulting bias between the two methods was 0.18 L/min with limits of agreement of -1.43 L/min to 1.78 L/min. Finally, Dey and Sprivulis developed and tested a protocol to optimize inter-assessor reliability with the USCOM^® ^device [29]. Two trained physicians performed blinded assessments on 21 emergency department patients. The inter-assessor correlation coefficient for CO measurements was 0.91 (95% CI = 0.85 to 0.95, *P *< 0.001), and the average difference between paired readings was 0.2 ± 0.2 L/min.

In the largest similar study to date, Monnet and colleagues studied 71 mechanically ventilated patients with an esophageal Doppler monitor in place [18]. An increase in aortic blood flow of 10% or more during PLR was found to predict volume response with a sensitivity of 97% and specificity of 94%. Boulain and colleagues studied 39 patients with a PAC and radial arterial line in place, and found that the change in pulse pressure and SV were significantly correlated both during PLR and following VE [30]. Lafanechère and colleagues examined 22 intubated and fully sedated patients with an esophageal Doppler monitor in place [31]. An increase in aortic blood flow of more than 8% during PLR predicted volume response with a sensitivity of 90% and specificity of 83%. Finally, Monnet and colleagues studied 34 mechanically ventilated patients with arterial lines in place who were not necessarily fully accommodated to the ventilator [32]. Changes in arterial pulse pressure and pulse contour-derived cardiac index during end-expiratory occlusion of the ventilator as well as changes in cardiac index during PLR were examined. An increase in cardiac index of 10% or more during PLR predicted an increase in cardiac index following VE of 15% or more with a sensitivity of 91% and a specificity of 100%. Changes in pulse pressure and cardiac index during end-expiratory occlusion had similar predictive value.

Our specificity is comparable with these studies, but our sensitivity is somewhat lower. This may be the result of a less selected patient population and the inclusion of patients regardless of underlying diagnoses that may diminish the effect of PLR. Included in our study was one patient with lower extremity contractures, two patients with extensive bilateral lower extremity deep venous thrombosis, two chronically bed-bound quadriplegic patients, two patients with unilateral below the knee amputation, one patient with massive ascites, and one patient with abdominal compartment syndrome. Additionally, the use of a less invasive technique may have contributed to our lower sensitivity. Non-invasive measures of cardiac function have been previously studied in conjunction with PLR, and also demonstrated lower sensitivity for predicting the response to VE. For example, Lamia and colleagues and Maizel and colleagues studied 24 and 34 patients, respectively, with transthoracic echocardiography in conjunction with PLR [19,20]. Changes in CO and SV were predictive of volume response, but the sensitivities were somewhat lower at 77% and 69%, respectively.

The dilemma of which patients to subject to VE is encountered daily in the ICU. One of the principal uses for the PAC was to differentiate between various etiologies of hypotension and thereby guide therapy to optimize a patients' hemodynamic status [33]. However, with numerous clinical trials showing no benefit, concerns about safety, and rampant misinterpretation of data, the PAC is being used infrequently now in North American ICUs. This is likely to be contributing to a situation of probable under-monitoring of many critically ill patients [34-39]. Many intensivists now base most of their VE decisions on the CVP [2,40]. However, the CVP is a poor predictor of volume responsiveness and should not be used to make clinical decisions regarding fluid management [10,41]. This underscores the need for alternative fluid management strategies.

This study has some limitations. First, there were 13 additional patients that were to be enrolled, but were either unable to perform PLR or an adequate Doppler signal could not be obtained. However, analgesia or sedation may have facilitated successful measurements in many of these patients. Second, the majority of patients enrolled in our study had sepsis or hypovolemia as the etiology of their circulatory insufficiency. This may limit somewhat the applicability of this technique. Third, there was a significant difference in the presence of arrhythmias between the groups of responders and non-responders. This clouds the issue of whether or not this technique can be employed in patients with arrhythmia. However, the SV change with PLR predicted the correct SV response to VE in 16 of the 18 patients with arrhythmia.

Finally, the use of repeat studies on the same patient as independent observations may have impacted the results of the analysis. It is possible that sequential measurements taken on the same patient were correlated, which could alter the error term for any given analysis. However, the patients enrolled in this study were being actively treated in the ICU, and repeat studies on the same patient were separated in time by at least 24 hours. Hemodynamic interventions performed in that time would presumably impact the results of subsequent studies, minimizing any correlation that may exist between the two studies. In support of this assertion, a limited analysis was repeated using only the first challenge on each patient, with results similar to those for the complete data set. The SV increased in response to PLR in the responders and non-responders by 21.7 ± 12.7% and 3.2 ± 12.0%, respectively (*P *< 0.001). The SV increased in response to VE in the responders and non-responders by 26.3 ± 13.3% and 2.0 ± 8.5%, respectively (*P *< 0.001). A SV increase induced by PLR of 15% or more predicted volume response with a sensitivity of 79%, specificity of 91%, positive predictive value of 90%, and a negative predictive value of 82%. The upper and lower limits of agreement in the repeatability analysis were 14.4% and -11.2%, respectively.

## Conclusions

We have demonstrated that a transthoracic Doppler ultrasound device can be used in conjunction with PLR to predict volume responsiveness in a variety of unselected medical ICU patients. Less than 50% of the patients subjected to fluid loading were volume responsive, underscoring the need for routine application of such methods when VE is considered. As with many non-invasive diagnostic maneuvers, results from this technique are likely best interpreted and clinically applied as one part of a larger clinical picture with the ultimate goal being a decrease in the amount of fluid loading that does not result in improved cardiac output.

## Key messages

• Non-invasive stroke volume measurement using transthoracic ultrasound can be utilized to determine fluid responsiveness in critically ill patients.

• Stroke volume changes in response to PLR correlate well with fluid challenges as a predictor of fluid responsiveness in critically ill patients.

• CVP measurements do not accurately reflect fluid responsiveness in critically ill patients.

• Less than 50% of ICU patients given fluid boluses are volume responsive.

## Abbreviations

CI: confidence interval; CO: cardiac output; CVP: central venous pressure; FTc: corrected flow time; ICU: intensive care unit; MAP: mean arterial pressure; PAC: pulmonary artery catheter; PLR: passive leg raise; ROC: receiver operating characteristic; SV: stroke volume; VE: volume expansion.

## Competing interests

The authors declare that they have no competing interests.

## Authors' contributions

WI conceived and designed the study, participated in drafting the manuscript, and provided supervision. MK participated in the study design, provided critical revision of the manuscript, and provided supervision. ST performed data acquisition, participated in drafting the manuscript, and performed statistical analysis. WI had full access to all of the data in the study and takes responsibility for the integrity of the data and the accuracy of the data analysis.
